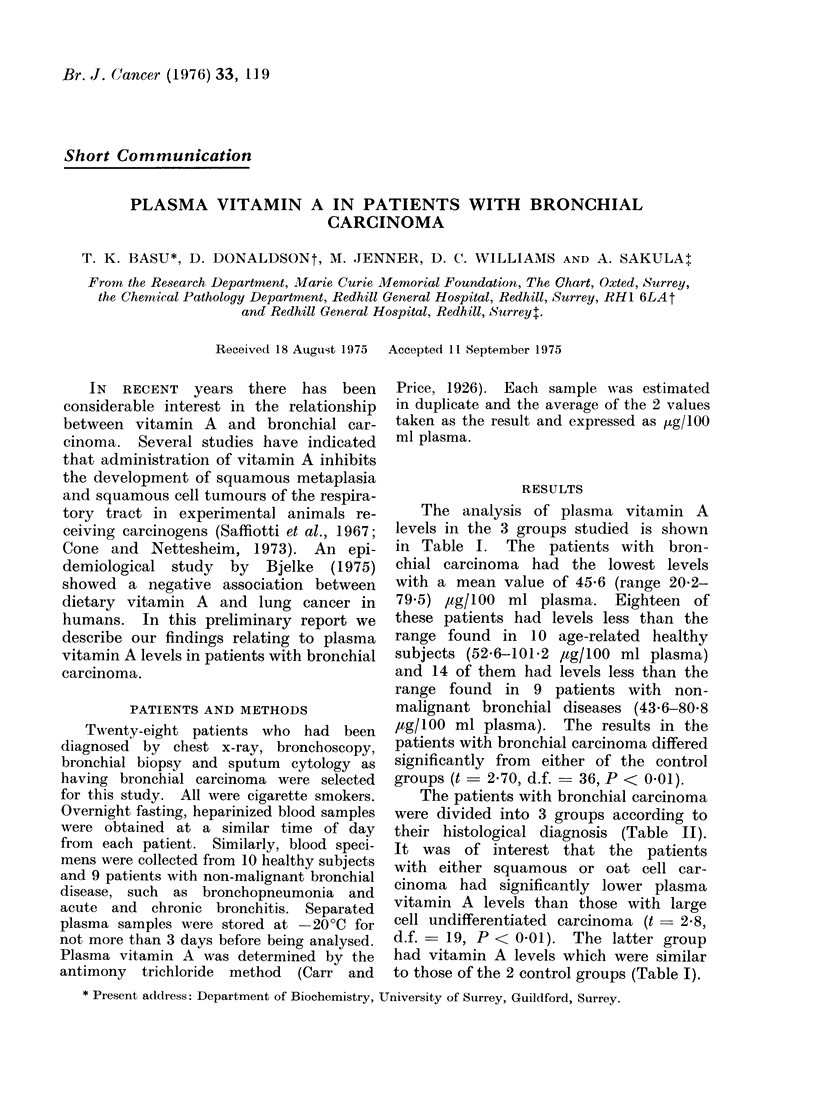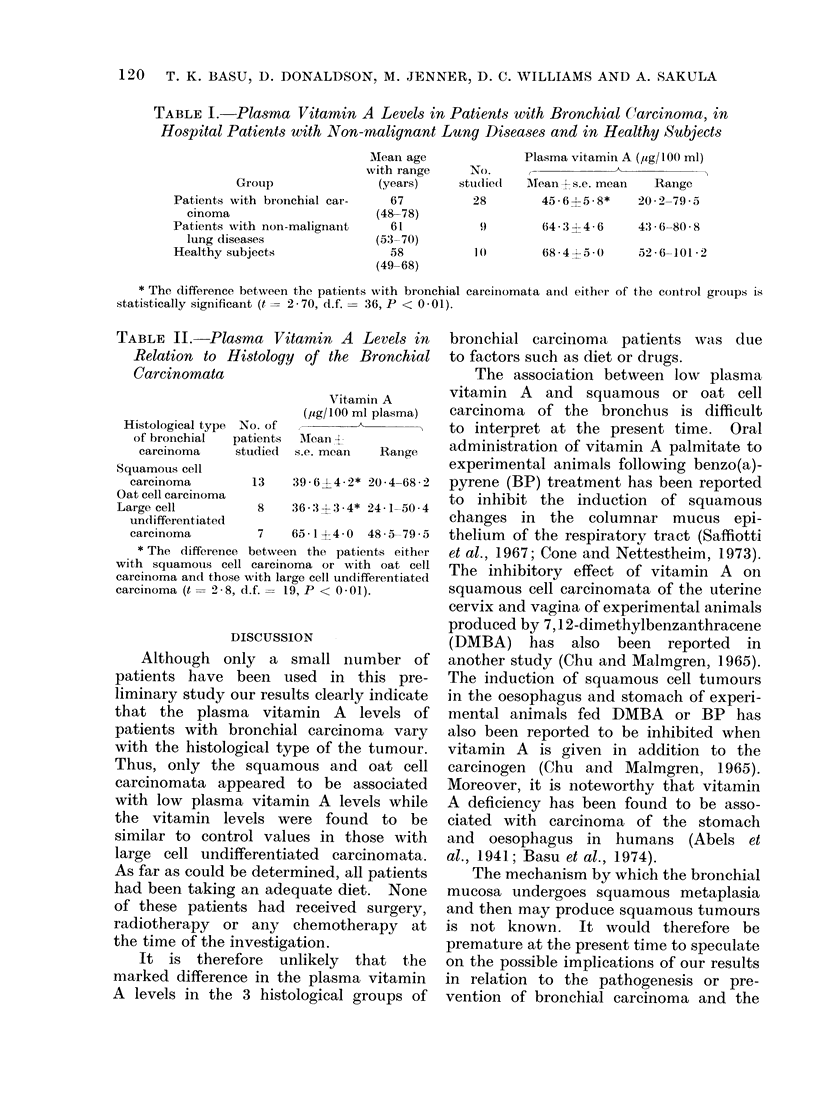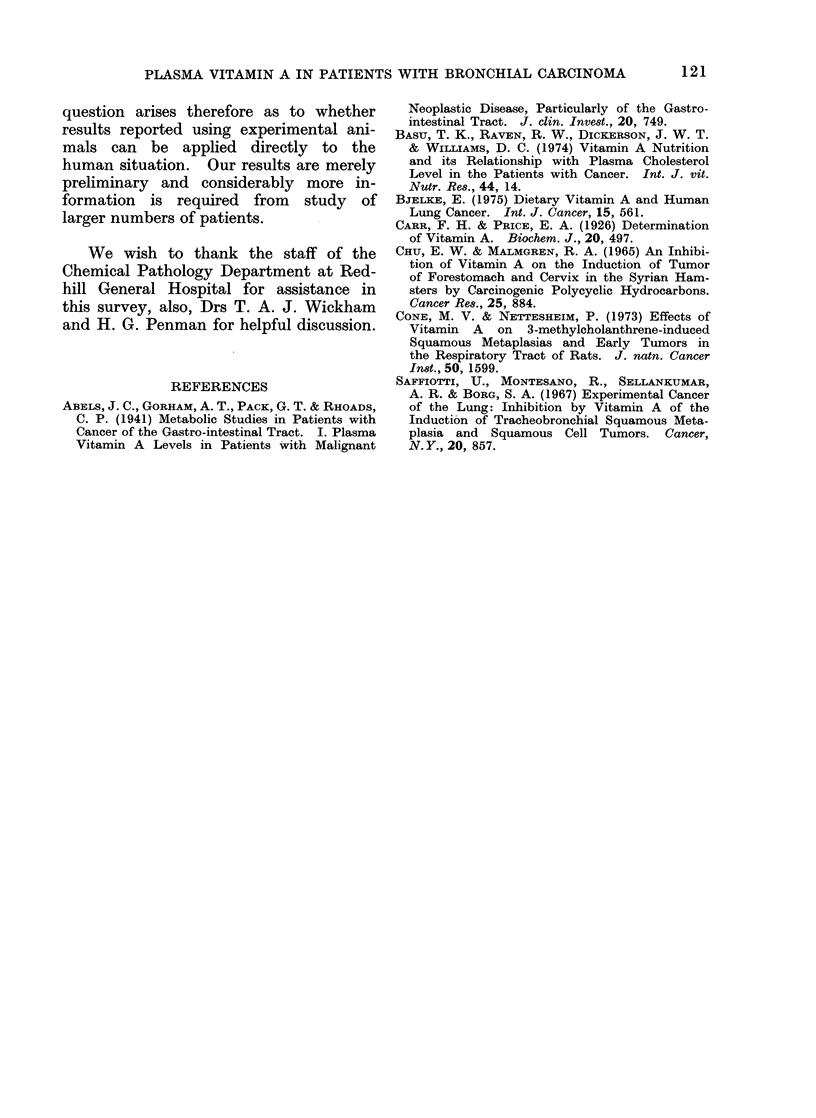# Plasma vitamin A in patients with bronchial carcinoma.

**DOI:** 10.1038/bjc.1976.14

**Published:** 1976-01

**Authors:** T. K. Basu, D. Donaldson, M. Jenner, D. C. Williams, A. Sakula


*Br. J. Cancer* (1976) **33**, 119

### *Short Communication*

### PLASMA VITAMIN A IN PATIENTS WITH BRONCHIAL CARCINOMA

T. K. BASU*, D. DONALDSON†, M. JENNER, D. C. WILLIAMS AND A. SAKULA‡

*From the Research Department, Marie Curie Memorial Foundation, The Chart, Oxted, Surrey, the Chemical Pathology Department, Redhill General Hospital, Redhill, Surrey, RH1 6LA† and Redhill General Hospital, Redhill, Surrey‡.*

Received 18 August 1975 Accepted 11 September 1975

IN RECENT years there has been considerable interest in the relationship between vitamin A and bronchial carcinoma. Several studies have indicated that administration of vitamin A inhibits the development of squamous metaplasia and squamous cell tumours of the respiratory tract in experimental animals receiving carcinogens (Saffiotti *et al.*, 1967; Cone and Nettesheim, 1973). An epidemiological study by Bjelke (1975) showed a negative association between dietary vitamin A and lung cancer in humans. In this preliminary report we describe our findings relating to plasma vitamin A levels in patients with bronchial carcinoma.

### PATIENTS AND METHODS

Twenty-eight patients who had been diagnosed by chest x-ray, bronchoscopy, bronchial biopsy and sputum cytology as having bronchial carcinoma were selected for this study. All were cigarette smokers. Overnight fasting, heparinized blood samples were obtained at a similar time of day from each patient. Similarly, blood specimens were collected from 10 healthy subjects and 9 patients with non-malignant bronchial disease, such as bronchopneumonia and acute and chronic bronchitis. Separated plasma samples were stored at $-20°C$ for not more than 3 days before being analysed. Plasma vitamin A was determined by the antimony trichloride method (Carr and Price, 1926). Each sample was estimated in duplicate and the average of the 2 values taken as the result and expressed as $\mu g/100$ ml plasma.

### RESULTS

The analysis of plasma vitamin A levels in the 3 groups studied is shown in Table I. The patients with bronchial carcinoma had the lowest levels with a mean value of 45·6 (range 20·2–79·5) $\mu g/100$ ml plasma. Eighteen of these patients had levels less than the range found in 10 age-related healthy subjects (52·6–101·2 $\mu g/100$ ml plasma) and 14 of them had levels less than the range found in 9 patients with non-malignant bronchial diseases (43·6–80·8 $\mu g/100$ ml plasma). The results in the patients with bronchial carcinoma differed significantly from either of the control groups ($t = 2·70$, d.f. $= 36$, $P < 0·01$).

The patients with bronchial carcinoma were divided into 3 groups according to their histological diagnosis (Table II). It was of interest that the patients with either squamous or oat cell carcinoma had significantly lower plasma vitamin A levels than those with large cell undifferentiated carcinoma ($t = 2·8$, d.f. $= 19$, $P < 0·01$). The latter group had vitamin A levels which were similar to those of the 2 control groups (Table I).

* Present address: Department of Biochemistry, University of Surrey, Guildford, Surrey.

TABLE I.—*Plasma Vitamin A Levels in Patients with Bronchial Carcinoma, in Hospital Patients with Non-malignant Lung Diseases and in Healthy Subjects*

| Group | Mean age with range (years) | No. studied | Plasma vitamin A (μg/100 ml) Mean ± s.e. mean | Range |
|---|---|---|---|---|
| Patients with bronchial carcinoma | 67 (48–78) | 28 | 45·6±5·8* | 20·2–79·5 |
| Patients with non-malignant lung diseases | 61 (53–70) | 9 | 64·3±4·6 | 43·6–80·8 |
| Healthy subjects | 58 (49–68) | 10 | 68·4±5·0 | 52·6–101·2 |

* The difference between the patients with bronchial carcinomata and either of the control groups is statistically significant ($t = 2 \cdot 70$, d.f. $= 36$, $P < 0 \cdot 01$).

TABLE II.—*Plasma Vitamin A Levels in Relation to Histology of the Bronchial Carcinomata*

| Histological type of bronchial carcinoma | No. of patients studied | Vitamin A (μg/100 ml plasma) Mean ± s.e. mean | Range |
|---|---|---|---|
| Squamous cell carcinoma | 13 | 39·6±4·2* | 20·4–68·2 |
| Oat cell carcinoma | 8 | 36·3±3·4* | 24·1–50·4 |
| Large cell undifferentiated carcinoma | 7 | 65·1±4·0 | 48·5–79·5 |

* The difference between the patients either with squamous cell carcinoma or with oat cell carcinoma and those with large cell undifferentiated carcinoma ($t = 2 \cdot 8$, d.f. $= 19$, $P < 0 \cdot 01$).

### DISCUSSION

Although only a small number of patients have been used in this preliminary study our results clearly indicate that the plasma vitamin A levels of patients with bronchial carcinoma vary with the histological type of the tumour. Thus, only the squamous and oat cell carcinomata appeared to be associated with low plasma vitamin A levels while the vitamin levels were found to be similar to control values in those with large cell undifferentiated carcinomata. As far as could be determined, all patients had been taking an adequate diet. None of these patients had received surgery, radiotherapy or any chemotherapy at the time of the investigation.

It is therefore unlikely that the marked difference in the plasma vitamin A levels in the 3 histological groups of bronchial carcinoma patients was due to factors such as diet or drugs.

The association between low plasma vitamin A and squamous or oat cell carcinoma of the bronchus is difficult to interpret at the present time. Oral administration of vitamin A palmitate to experimental animals following benzo(a)-pyrene (BP) treatment has been reported to inhibit the induction of squamous changes in the columnar mucus epithelium of the respiratory tract (Saffiotti *et al.*, 1967; Cone and Nettestheim, 1973). The inhibitory effect of vitamin A on squamous cell carcinomata of the uterine cervix and vagina of experimental animals produced by 7,12-dimethylbenzanthracene (DMBA) has also been reported in another study (Chu and Malmgren, 1965). The induction of squamous cell tumours in the oesophagus and stomach of experimental animals fed DMBA or BP has also been reported to be inhibited when vitamin A is given in addition to the carcinogen (Chu and Malmgren, 1965). Moreover, it is noteworthy that vitamin A deficiency has been found to be associated with carcinoma of the stomach and oesophagus in humans (Abels *et al.*, 1941; Basu *et al.*, 1974).

The mechanism by which the bronchial mucosa undergoes squamous metaplasia and then may produce squamous tumours is not known. It would therefore be premature at the present time to speculate on the possible implications of our results in relation to the pathogenesis or prevention of bronchial carcinoma and the question arises therefore as to whether results reported using experimental animals can be applied directly to the human situation. Our results are merely preliminary and considerably more information is required from study of larger numbers of patients.

We wish to thank the staff of the Chemical Pathology Department at Redhill General Hospital for assistance in this survey, also, Drs T. A. J. Wickham and H. G. Penman for helpful discussion.

### REFERENCES

ABELS, J. C., GORHAM, A. T., PACK, G. T. & RHOADS, C. P. (1941) Metabolic Studies in Patients with Cancer of the Gastro-intestinal Tract. I. Plasma Vitamin A Levels in Patients with Malignant Neoplastic Disease, Particularly of the Gastro-intestinal Tract. *J. clin. Invest.*, **20**, 749.

BASU, T. K., RAVEN, R. W., DICKERSON, J. W. T. & WILLIAMS, D. C. (1974) Vitamin A Nutrition and its Relationship with Plasma Cholesterol Level in the Patients with Cancer. *Int. J. vit. Nutr. Res.*, **44**, 14.

BJELKE, E. (1975) Dietary Vitamin A and Human Lung Cancer. *Int. J. Cancer*, **15**, 561.

CARR, F. H. & PRICE, E. A. (1926) Determination of Vitamin A. *Biochem. J.*, **20**, 497.

CHU, E. W. & MALMGREN, R. A. (1965) An Inhibition of Vitamin A on the Induction of Tumor of Forestomach and Cervix in the Syrian Hamsters by Carcinogenic Polycyclic Hydrocarbons. *Cancer Res.*, **25**, 884.

CONE, M. V. & NETTESHEIM, P. (1973) Effects of Vitamin A on 3-methylcholanthrene-induced Squamous Metaplasias and Early Tumors in the Respiratory Tract of Rats. *J. natn. Cancer Inst.*, **50**, 1599.

SAFFIOTTI, U., MONTESANO, R., SELLANKUMAR, A. R. & BORG, S. A. (1967) Experimental Cancer of the Lung: Inhibition by Vitamin A of the Induction of Tracheobronchial Squamous Metaplasia and Squamous Cell Tumors. *Cancer, N.Y.*, **20**, 857.